# Impact of exercise and leucine-enriched protein supplementation on physical function, body composition, and inflammation in pre-frail older adults: a quasi-experimental study

**DOI:** 10.3389/fmed.2023.1204198

**Published:** 2023-08-14

**Authors:** Reshma Aziz Merchant, Yiong Huak Chan, Denishkrshna Anbarasan, Santhosh Seetharaman, Lydia Au, Vidhya Nachammai, Alexa Lai, Vanda Ho, Beatrix Ling Ling Wong, Eunice Pang, Kalpana Bhaskaran

**Affiliations:** ^1^Division of Geriatric Medicine, Department of Medicine, National University Hospital, Singapore, Singapore; ^2^Department of Medicine, National University Singapore, Singapore, Singapore; ^3^Biostatistics Unit, Yong Loo Lin School of Medicine, National University of Singapore, Singapore, Singapore; ^4^Healthy Ageing Programme, Alexandra Hospital, National University Health System, Singapore, Singapore; ^5^Department of Geriatrics Medicine, Ng Teng Fong General Hospital, Singapore, Singapore; ^6^Health Promotion Board, Singapore, Singapore; ^7^Glycemic Index Research Unit, School of Applied Science, Temasek Polytechnic, Singapore, Singapore

**Keywords:** leucine-enriched protein supplement, pre-frail, tumor necrosis factor alpha, interleukin-6, body composition, physical function

## Abstract

**Background:**

Exercise and a protein-enriched diet are essential for muscle protein synthesis, cellular growth, mitochondrial function, and immune function. The U.S. Food and Nutrition Board's current guideline on recommended dietary allowance for protein in older adults is 0.8 g/kg per day, which may not be sufficient in vulnerable pre-frail older adults.

**Aims:**

This study aimed to evaluate the impact of leucine-enriched protein supplementation with or without exercise over 3 months in pre-frail older adults who consumed ≤1 g/kg/day of protein on improving (i) physical function, (ii) body composition measures, and (iii) inflammatory biomarkers such as interleukin-6 (IL-6) and tumor necrosis factor alpha (TNF-α).

**Methods:**

A non-randomized cluster quasi-experimental study guided by the Strengthening the Reporting of Observational Studies in Epidemiology (STROBE) checklist of 178 pre-frail older adults [112 control, 44 nutrition (Nu), and 22 in the nutrition with exercise (Nu+Ex) group] comparing the effect of Nu+Ex and Nu on physical function, body composition, and inflammation. At 0, 3, and 6 months, questionnaires on demographics, depression, perceived health, and cognition were administered. Physical function assessment (short physical performance battery [SPPB] test, gait speed, handgrip strength, 5× sit-to-stand [STS]) was conducted, and body composition analysis was performed using a bioelectrical impedance analysis machine. IL-6 and TNF-α were measured at 0 and 3 months.

**Results:**

At 3 months, there were significant improvements in gait speed, 5× STS, SPPB scores, depression, perceived health, fat-free mass, and appendicular skeletal muscle mass indices in the Nu+Ex group. Both Nu+Ex and Nu groups had improvements in body cell mass and reductions in IL-6 and TNF-α. The improvements were not sustained after 6 months.

**Conclusion:**

Our study results need to be validated in future longitudinal randomized studies with a larger sample size focusing on populations at risk.

## 1. Introduction

The world's population is aging at an unprecedented rate, especially in the Asia-Pacific region, where countries such as Singapore and South Korea have the fastest aging population (1). Worldwide, the number of older adults 65 years of age and older is expected to double to 1.5 billion by 2050 and may account for 33.3% of the population in Singapore during the same time frame (2, 3). Aging is associated with declines in physical function, anorexia of aging, sarcopenia, and frailty, which are major contributors to healthcare costs (4). Frailty is a state of declining physiological reserve that predisposes older adults to an increased risk of adverse outcomes, while sarcopenia is defined as a loss of muscle function, quality, or mass. It is a component of physical frailty (5, 6). Pre-frail is a transition phase to frailty, where one in five may progress to frailty over 3 years. The progression is associated with double the healthcare cost, and pre-frailty may be reversible before the onset of disability (4, 7). The combination of a sedentary lifestyle, increased prevalence of multimorbidity, and low protein and energy intake secondary to anorexia of aging are well-recognized factors contributing to the loss of muscle mass, frailty, and disability in older adults (8). Nutrition and exercise are well-recognized modifiable factors for a longer health span, especially in sarcopenic, and frail older adults. There are multiple factors associated with low energy and protein intake in older adults, such as poor oral health, multimorbidity with superimposed dietary restriction, limited access to food, financial insecurity, polypharmacy, and dysregulation of gut peptide releases such as ghrelin, leptin, and insulin (9, 10).

Protein is a vital nutrient in the diet of older adults. It is responsible for maintaining good oral health, musculoskeletal function, immune function, wound healing, and improving insulin sensitivity (9). Leucine is a branched-chain amino acid, and leucine-enriched protein supplements, together with resistance exercise, have been shown to potentiate muscle protein synthesis, reduce anabolic resistance, and improve muscle mass and physical function in healthy and sarcopenic older adults (11, 12). Since the COVID-19 pandemic, the role of a protein-enriched diet and exercise has become even more important, specifically to restore muscle mass loss caused by lockdown and movement restrictions (13). The U.S. Food and Nutrition Board's current guideline on the recommended dietary allowance (RDA) for protein in older adults is 0.8 g/kg per day. However, it is well known that older adults require much higher protein than the RDA due to body composition changes, low-grade inflammation, multimorbidity, insulin resistance, and a higher propensity to develop anabolic resistance (14–16).

A high protein diet supplemented by leucine and/or vitamin D has been shown to reduce proinflammatory biomarkers such as chemerin and progranulin in diabetics and attenuate the rise of interleukin-6 (IL-6) in sarcopenic older adults (17, 18). The role of resistance exercise in combination with leucine-enriched protein supplementation in improving muscle mass, physical function, and inflammatory biomarkers such as IL-6 and tumor necrosis factor-alpha (TNF-α) has shown mixed results in frail or pre-frail older adults who are at greatest risk of functional decline (19). Most prior studies have either focused on healthy or sarcopenic older adults, and there are limited studies on lower baseline protein consumption as inclusion criteria. This study aimed to evaluate if a diet enriched with an additional 16 g of protein and 3 g of leucine supplementation with or without exercise over 3 months in pre-frail older adults who consume ≤ 1 g/kg of protein a day improves (i) physical function, (ii) fat-free mass (FFM) and appendicular skeletal muscle (ASM) mass, and (iii) systemic inflammation as measured by IL-6 and TNF-α.

## 2. Materials and methods

### 2.1. Participants and study design

This study was originally designed as a three-cluster randomized control trial but had to be converted to a non-randomized quasi-experimental design due to COVID-19 restrictions and lockdown. The initial sample size calculations of at least 65 participants per group were based on 80% power and two-sided 5% significance on a conservative Interclass Correlation Coefficient (ICC) of 0.02 and a Cohen's effect size of 0.5 in the gait speed and muscle mass differences between the three groups. The important modifications in response to extenuating circumstances were approved by the local ethics board and did not affect the research question or outcomes (20). It impacted mainly the change in the control population, which was part of another study with similar inclusion criteria. The Strengthening the Reporting of Observational Studies in Epidemiology (STROBE) checklist for observational studies was used to guide the reporting of the findings (21, 22), and the baseline differences were adjusted for in the final analysis.

The inclusion criteria included participants ≥60 years old, able to provide consent and adhere to instructions as deemed suitable by a primary care physician or trained members of the study team, ambulant, and pre-frail. Participation was entirely voluntary. Participants with a pacemaker or a defibrillator, liver or gastrointestinal disease, end-stage lung disease, cardiac disease, cancer undergoing active treatment, gout or psychiatric conditions, and nursing home residents were excluded. Since this study included a nutritional component, participants with creatinine clearance < 30 ml/min, HbA1c >8.0%, food allergies or intolerances, nutrition therapy or precaution feeding, and baseline protein intake exceeding 1 g/kg body weight/day were also excluded.

**Figure 1:**
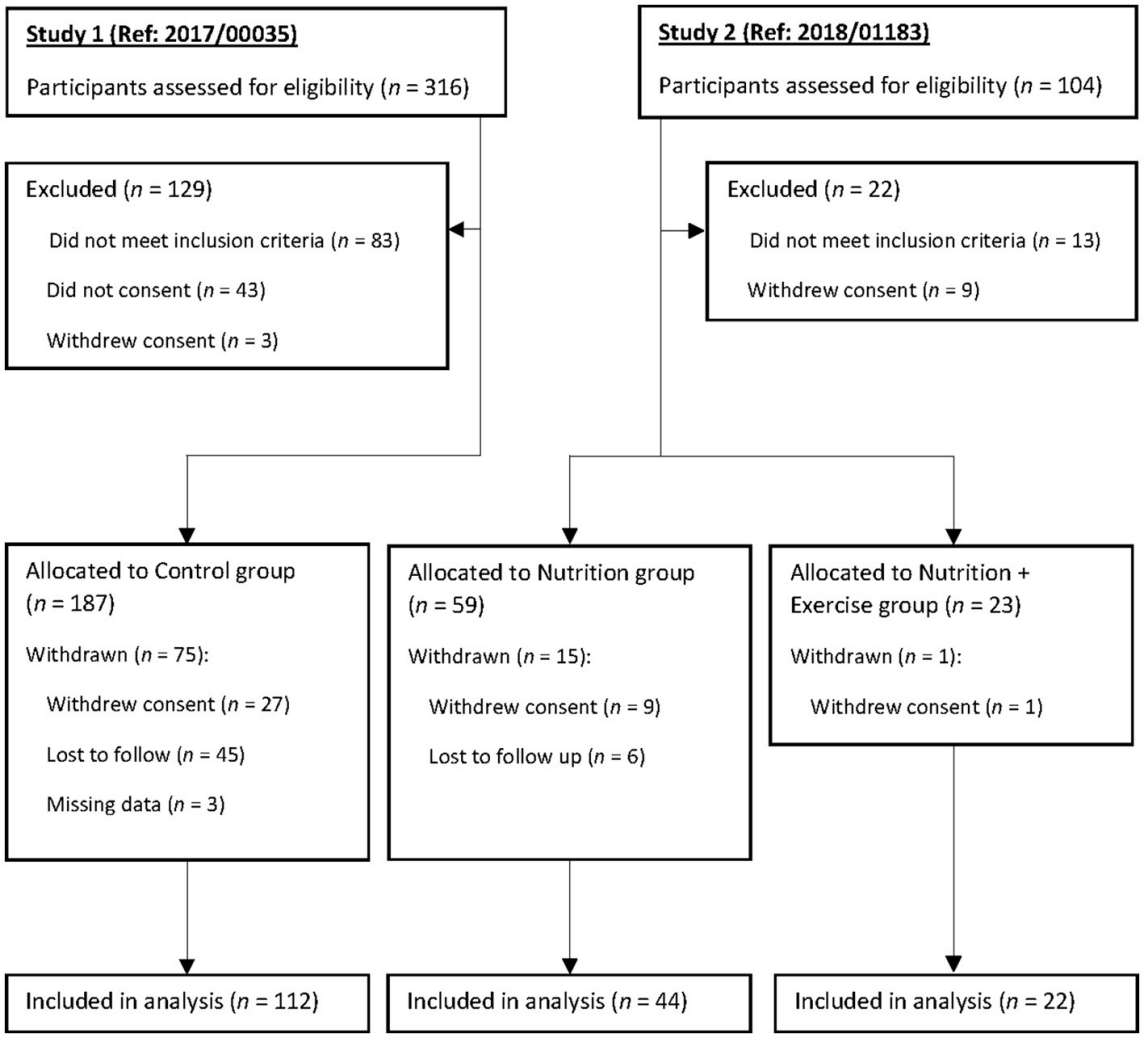
Participant screening, group allocation, and follow-up. The per-protocol population consisted of participants who completed the 3-month intervention.

The intervention was for the duration of 3 months, with a further follow-up of 3 months after the discontinuation of the intervention for a total of 6 months. The intervention groups received either nutrition (Nu) only (an average of 16 g of protein and 3 g of leucine) or nutrition and biweekly 60-min exercise comprising strength, balance, and resistance training (nutrition with exercise [Nu+Ex]). Both the Nu and Nu+Ex groups were recruited from the National University Hospital ambulatory care clinics and community (i.e., senior activity centers and community centers). Due to the COVID-19 lockdown and the research team's movement restrictions, a comparison control group was recruited from the Choa Chu Kang National University Polyclinic, a primary care center located within a housing estate in the Western part of Singapore (Figure 1). A total of 269 participants (Figure 1) were enrolled in the study: 82 in the intervention group (Nu = 59; Nu+Ex = 23) and 187 controls, with complete data at 6 months available for 66 in the intervention group (Nu = 44; Nu+Ex = 22) and 112 controls. The allocations to groups were based on ongoing COVID-19 restrictions, e.g., when no group exercises were permitted, participants received nutrition interventions. Furthermore, due to the pandemic, many participants were hesitant to attend follow-up assessments and hence missed them, contributing to the number of individuals lost to follow-up. Therefore, only participants with complete data were included in the final analysis.

### 2.2. Procedures

During recruitment, three 24-h dietary recalls (2 weekdays and 1 weekend) were conducted to ensure participants' baseline protein intake was ≤ 1 g/kg body weight/day. Dietary recall data were coded and input into FoodWorks Premium Edition (Version 10, Xyris Software, Brisbane, Australia, 2019) (23). Similar to FoodWorks, a database of Australian and New Zealand foods, nutritional information for food items from Singapore could be obtained directly from nutrition labels on the food items or local databases, such as the Singapore Health Promotion Board's Energy and Nutrient Composition of Food database (24). Protein intake per body weight was calculated by dividing the mean daily protein intake by body weight.

All participants in the Nu and Nu+Ex groups were provided with leucine-enriched protein supplements, predominantly whey and soy protein combination, in the form of specially created local foods or beverages such as granola, coffee, chicken porridge, or specially manufactured beverage powders that are not available for sale locally. Participants received an average of 16 g of protein and 3 g of leucine daily for 3 months. Once a month, study team members contacted participants to monitor compliance and adverse outcomes and obtain three 24-h dietary recalls (2 weekdays and 1 weekend). Participants in the Nu+Ex group were additionally provided biweekly exercise sessions lasting 60 min, each session comprising strength, balance, and resistance exercises using resistant bands conducted by physiotherapists. The control group only received health education advice. The study lasted for 6 months, with participants being assessed at baseline, 3 months, and 6 months post-baseline.

### 2.3. Demographics and covariates

A combined interview questionnaire consisting of demographics, chronic diseases, medications, perceived health, physical activity and function, depression, frailty, sarcopenia, falls, cognition, and nutrition was administered by trained study team members. Polypharmacy was defined as consuming ≥5 types of medications daily. The FRAIL scale (Fatigue, Resistance, Aerobic, Illness, and Loss of Weight) was used to assess frailty, where pre-frail was defined as 1–2 with a maximum score of 5 (25, 26). The FRAIL scale is based on a 5-item questionnaire and has been validated in many countries across the world, including Singapore (27). The Rapid Physical Assessment (RAPA) tool was used to assess physical activity (28). Katz's activity of daily living (ADL) scale was used to assess ADL, and Lawton's instrumental activities of daily living (IADL) scale was used to assess IADL (29, 30). Participants were classified as fallers if they experienced one or more falls in the past year (31). The Montreal Cognitive Assessment (MoCA) was used to assess cognition (32). Depression was evaluated using the 15-item Geriatric Depression Scale (GDS-15), where scores >5 indicated depression (33). The Mini Nutritional Assessment Short-Form (MNA-SF) evaluated the risk of malnutrition (34).

### 2.4. Physical performance

Physical performance measures comprised maximum handgrip strength (HGS), gait speed, and the short physical performance battery (SPPB) test. HGS was measured using the Jamar hand dynamometer on the dominant hand with the elbow flexed at 90° in the seated position. The SPPB includes three components—balance, gait speed, and 5× sit-to-stand (STS)—and is scored out of 12 points (4 points per component). A gait speed of < 1.0 m/s was considered slow.

### 2.5. Body composition

Body mass index (BMI) was calculated by dividing body weight (kg) by height squared (m^2^). Body composition was measured using the InBody S10 multi-frequency bioelectrical impedance analyzer (BIA), which included measures of body fat percentage (BF%), fat mass (FM), FFM, ASM, body cell mass, and phase angle. Fat mass index (FMI), fat-free mass index (FFMI), and appendicular skeletal muscle index (ASMI) were obtained by dividing FM, FFM, and ASM by height squared, respectively. Sarcopenia diagnosis was based on the 2019 Asian Working Group for Sarcopenia (AWGS) criteria of gender-specific cutoffs for ASMI and either low HGS (< 28 kg for men and <18 kg for women) or low physical performance (35).

### 2.6. Inflammatory biomarkers

The TNF-α and IL-6 inflammatory biomarkers were measured by an accredited hospital-based laboratory at 0 and 3 months in a subgroup of participants who agreed to have their blood drawn. TNF-α was measured using immunoenzymetric assays with a detection range between 1.0 and 498 pg/mL, and IL-6 was measured using the electrochemiluminescence immunoassay (ECLIA) with a detection range between 1.5 and 50,000 pg/mL.

### 2.7. Statistical analysis

IBM SPSS Version 28.0 was used to analyze our data. Per-protocol analysis was performed for participants with a complete set of data. Categorical variables were presented as frequencies with percentages, while continuous variables were presented as mean ± standard deviation when normality assumptions were satisfied (checked using the Shapiro–Wilk test); otherwise, medians with the interquartile range (IQR) were shown. Significance testing of categorical variables was assessed using the chi-square of Fisher's exact tests. Significance testing for normally distributed variables was carried out using a one-way ANOVA; otherwise, the Kruskal–Wallis test was used.

General linear model (GLM) and quantile regression were used to compare changes in normally distributed continuous and non-normally distributed variables between groups, respectively, adjusting for age, sex, ethnicity, years of education, BMI, hypertension, hyperlipidemia, diabetes, polypharmacy, sarcopenia, protein intake, intervention compliance, and corresponding values from the preceding time point.

Plasma biomarker values are presented as median (IQR) in Table 2. Mood's median test was used for significance testing at baseline, while quantile regression was used to compare changes between groups with the control group as the reference. The regression was further adjusted for age, sex, ethnicity, years of education, BMI, hypertension, hyperlipidemia, diabetes, polypharmacy, sarcopenia, protein intake, intervention compliance, and corresponding baseline values.

### 2.8. Ethics approval and informed consent

This study conformed to the principles of the Declaration of Helsinki and was approved by the National Healthcare Group Domain Specific Review Board (Reference: 2018/01183 and 2019/00017). Informed consent was obtained from all participants.

## 3. Results

A total of 178 participants were included in the final analysis (Table 1). The mean age of the participants was 72.56 years. There were significantly more males in the control group (55.4%) than in the Nu (27.3%) and Nu+Ex (27.3%) groups. Men were generally reluctant to participate in interventions, which is a known phenomenon worldwide. There was a higher prevalence of the Chinese ethnic group in the Nu group (95.5%), followed by the Nu+Ex group (90.9%) and the control group (82.1%). The Indian and Malay ethnic minority groups were significantly higher in the control group (9.8 and 8.0%, respectively) compared with the Nu (2.3% each) and none in the Nu+Ex group. Diabetes prevalence based on self-reporting was significantly higher in the control group (53.6%) compared with the Nu (22.7%) and Nu+Ex groups (9.1%). This was also evident in the prevalence of hypertension and hyperlipidemia, which was the highest in the control group and the lowest in the Nu+Ex group.

**Table 1:** Baseline characteristics.

	**Control**	**Nutrition**	**Nutrition + Exercise**	***P*-value**
	*n =* 112 (62.9%)	*n =* 44 (24.7%)	*n =* 22 (12.4%)	
**Demographics**				
Age (years)	71.65 ± 5.06	74.57 ± 7.82	71.45 ± 7.07	0.081
**Sex**				**0.001**
Male	62 (55.4)	12 (27.3)	6 (27.3)	
Female	50 (44.6)	32 (72.7)	16 (72.7)	
**Ethnicity**				**0.032**
Chinese	92 (82.1)	42 (95.5)	20 (90.9)	
Malay	9 (8.0)	1 (2.3)	0 (0.0)	
Indian	11 (9.8)	1 (2.3)	0 (0.0)	
Others	0 (0.0)	0 (0.0)	1 (4.5)	
Hypertension	81 (72.3)	23 (52.3)	7 (31.8)	**< 0.001**
Hyperlipidemia	99 (88.4)	23 (52.3)	8 (36.4)	**< 0.001**
Diabetes	60 (53.6)	10 (22.7)	2 (9.1)	**< 0.001**
Polypharmacy	32 (28.6)	5 (11.4)	2 (9.1)	**0.018**
Living alone	8 (7.1)	9 (20.5)	3 (13.6)	0.056
BMI (kg/m^2^)	26.12 ± 4.51	23.07 ± 3.68	22.73 ± 3.62	**< 0.001**
Education (years)	7.45 ± 3.75	7.00 ± 4.34	11.32 ± 6.63	**0.023**
Perceived health (EQ-VAS)	70.01 ± 13.70	68.83 ± 13.21	73.55 ± 12.45	0.404
Physical activity (RAPA)	3.68 ± 1.52	3.05 ± 1.73	3.50 ± 1.37	**0.013**
At least 1 IADL impairment	9 (8.0)	3 (6.8)	1 (4.5)	0.839
At least 1 ADL impairment	19 (17.0)	5 (11.4)	1 (4.5)	0.260
Sarcopenia^*a*^	14 (12.5)	9 (20.5)	2 (9.1)	**< 0.001**
≥1 fall in 1 year	23 (20.5)	18 (40.9)	3 (13.6)	**0.014**
MoCA	25.69 ± 2.86	25.69 ± 3.90	26.19 ± 4.05	0.300
Cognitive impairment	47 (42.0)	17 (38.6)	9 (40.9)	0.930
Depression (GDS)	32 (28.6)	14 (31.8)	7 (31.8)	0.901
MNA-SF total	12.77 ± 1.71	12.50 ± 1.42	12.68 ± 1.09	0.164
**Nutritional status (MNA-SF)**				0.894
Malnourished	1 (0.9)	0 (0.0)	0 (0.0)	
At risk of malnourishment	19 (17.0)	9 (20.5)	3 (13.6)	
Normal nutritional status	92 (82.1)	35 (79.5)	19 (86.4)	
**Physical performance**				
Handgrip strength (kg)	23.60 ± 7.02	18.11 ± 5.65	21.16 ± 8.61	**< 0.001**
Gait speed (m/s)	0.94 ± 0.20	0.94 ± 0.33	1.18 ± 0.30	**0.003**
5× sit-to-stand time (s)	13.18 ± 5.11	14.25 ± 5.49	10.86 ± 2.35	**0.004**
Total SPPB score	9.86 ± 1.99	8.84 ± 2.51	11.05 ± 1.46	**< 0.001**
**Body composition**				
Body fat percentage (%)	33.62 ± 9.75	32.18 ± 7.69	25.22 ± 5.32	**< 0.001**
Fat mass index (kg/m^2^)	9.02 ± 3.79	7.58 ± 2.78	6.70 ± 2.95	**0.006**
Fat-free mass index (kg/m^2^)	16.89 ± 1.94	15.35 ± 1.78	16.24 ± 2.31	**< 0.001**
Fat mass/fat-free mass	0.54 ± 0.24	0.50 ± 0.17	0.42 ± 0.18	0.096
50 khz trunk phase angle (θ)	5.06 ± 0.65	4.41 ± 0.68	4.78 ± 0.63	**< 0.001**
Body cell mass (kg)	27.13 ± 6.28	22.74 ± 4.89	25.16 ± 4.80	**< 0.001**
ASMI (kg/m^2^)	6.90 ± 1.06	5.76 ± 1.15	6.19 ± 1.12	**< 0.001**

Values presented as *n* (%) or mean ± SD. BMI, body mass index; ADL, activities of daily living; MoCA, Montreal Cognitive Assessment; SPPB, short physical performance battery; ASMI, appendicular skeletal muscle index. ^*a*^Based on the Asian Working Group for Sarcopenia (AWGS) 2019's definition. Bold indicates significance (*p* < 0.05).

### 3.1. Physical function, cognition, depression, and perceived health

Baseline physical functions, such as gait speed, HGS, 5× STS, and total SPPB scores, were significantly different between the groups (Table 1). Gait speed was significantly lower in the control (0.94 m/s) and Nu groups (0.94 m/s) compared with the Nu+Ex group (1.18 m/s). The total SPPB score was significantly lower in the Nu group (8.84), followed by the control group (9.86) and the Nu+Ex group (11.05). Similarly, 5× STS time was significantly longer in the Nu group compared with the control and Nu+Ex groups at 14.3 s, 13.2 s, and 10.9 s, respectively. There were no significant differences in perceived health or depression at baseline between the groups.

**Figure 2:**
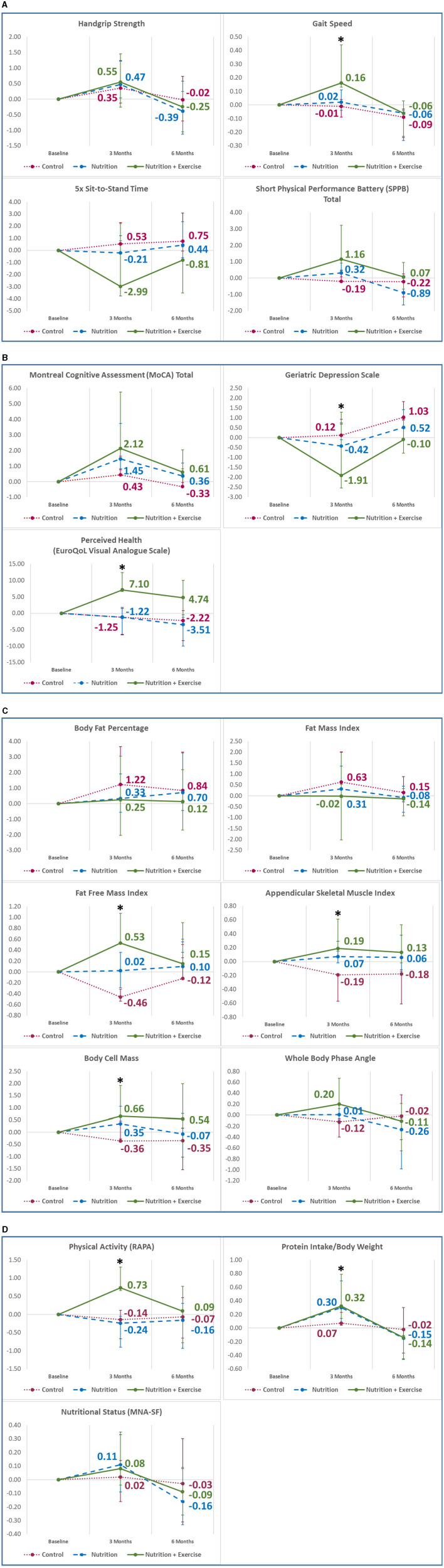
Mean changes in outcome variables from baseline to 6 months in **(A)** physical function; **(B)** cognition, depression, and perceived health; **(C)** body composition; **(D)** physical activity and nutritional information. Analysis adjusted for age, sex, ethnicity, education, body mass index, hypertension, hyperlipidemia, diabetes, polypharmacy, sarcopenia, protein intake, intervention compliance, and corresponding values from the preceding time point. Vertical bars indicate the 95% confidence interval. *p < 0.05; **p < 0.001; RAPA, rapid assessment of physical activity; MNA-SF, Mini Nutritional Assessment Short-Form.

Within the Nu+Ex group, there was a significant improvement in gait speed at 3 months (β 0.16, 95% CI 0.03–0.28; *p* = 0.005) (Figure 2A), depression as reflected by decreased GDS score (β −1.91, 95% CI −3.20 to −0.62) and improved perceived health (β 7.10, 95% CI 0.42–10.30; *p* = 0.036) (Figure 2B). At 6 months, there was a decline in both the Nu and Nu+Ex groups, with no significant difference between the groups (Figure 2).

**Figure 3:**
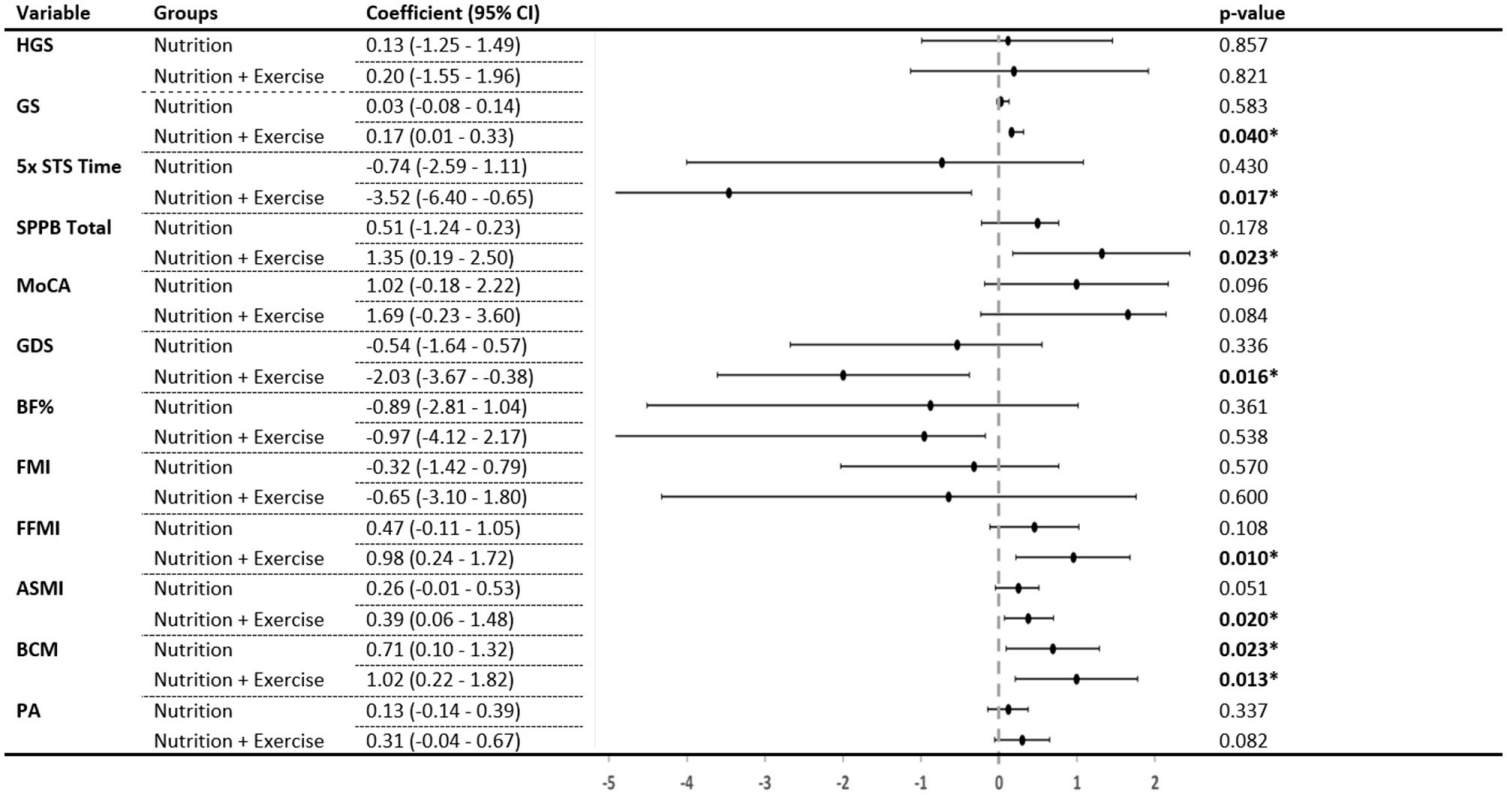
Change in each group compared with the control group after 3 months. *p*-values generated from a general linear model with age, sex, ethnicity, education, body mass index, hypertension, hyperlipidemia, diabetes, polypharmacy, sarcopenia, protein intake, intervention compliance, and corresponding values from the preceding time point as covariates. HGS, handgrip strength; GS, gait speed; 5× STS, 5× sit-to-stand; SPPB, short physical performance battery; MoCA, Montreal Cognitive Assessment; GDS, Geriatric Depression Scale; BF%, body fat percentage; FMI, fat mass index; FFMI, fat-free mass index; ASMI, appendicular skeletal muscle index; BCM, body cell mass; PA, whole-body phase angle. *Indicates significant difference (*p* < 0.05) when compared with the control group.

Compared with the control group and after adjustment for confounding factors and baseline measures, the Nu+Ex group improved significantly in gait speed (β 0.17, 95% CI 0.01–0.33; *p* = 0.040), 5× STS (β −3.52, 95% CI −6.40 to −0.65; *p* = 0.017), total SPPB scores (β 1.35, 0.19–2.50; *p* = 0.023), GDS scores (β −2.03, 95% CI −3.67 to −0.38) (Figure 3), and perceived health (β 8.35, 95% CI 1.41–12.50; *p* = 0.023) (Supplementary Table 1) at 3 months.

### 3.2. Body composition

Baseline FFMI, phase angle, body cell mass, and ASMI were significantly higher in the control group (16.89 kg, 5.06 kg, 27.13 kg, and 6.90 kg/m^2^) compared with the Nu+Ex (16.24 kg, 4.78 kg, 25.16 kg, and 6.19 kg/m^2^) and Nu groups (15.35 kg, 4.41 kg, 22.74 kg, and 5.76 kg/m^2^), respectively. Within the Nu+Ex group, there was significant improvement in FFMI (β 0.53, 95% CI 0.01–0.54; *p* = 0.029), ASMI (β 0.19, 95% CI 0.04–0.42; *p* = 0.037), and body cell mass (β 0.66, 95% CI 0.07–1.25; *p* = 0.039) (Figure 2C). At 6 months, there was a decline in FFMI, body cell mass, and appendicular skeletal mass index in the Nu and Nu+Ex groups, with no significant difference between the groups.

Compared with controls and after adjustment for confounding factors and baseline measures, the Nu+Ex group showed significant improvement in the FFMI (β 0.98, 95% CI 0.24–1.72; *p* = 0.010), ASMI (β 0.39, 95% CI 0.06–1.48; *p* = 0.020) and body cell mass (β 1.02, 95% CI 0.22–1.82; *p* = 0.013). The Nu group only showed improvement in body cell mass (β 0.71, 95% CI 0.10–1.32; *p* = 0.023) (Figure 3).

### 3.3. Inflammatory biomarkers

There were no significant differences in the baseline median levels of IL-6 and TNF-α between the groups (Table 2). However, at 3 months, there was a significant decrease in the IL-6 levels in the Nu+Ex group (β −4.32, 95% CI −5.31 to −3.05; *p* < 0.001) and the Nu group (β −8.24, 95% CI −9.05 to −7.19; *p* < 0.001) (Table 2). Similarly, there was a significant decrease in the TNF-α levels at 3 months in the Nu+Ex (β −2.10, 95% CI −3.26 to −1.01; *p* < 0.001) and Nu groups (β −1.88, 95% CI −2.94 to −0.81; *p* < 0.001).

**Table 2:** Unadjusted and adjusted quantile regression models of median change in plasma biomarkers after 3 months of intervention.

**Biomarker**	**Group**	**Baseline median (IQR)^#^**	**Unadjusted**	**Adjusted^+^**
		**Coefficient (95% CI)** ***p*****-value**	**Coefficient (95% CI)** ***p*****-value**
IL-6 (pg/mL)	Control	3.20 (1.80)	Reference	Reference
	Nutrition	2.80 (1.20)	−5.10 (−1.69 to 0.67) *p =* 0.383	–**8.24 (**–**9.05 to** –**7.19)** ***p** **<*** **0.001**
	Nutrition + Exercise	2.30 (0.80)	−0.20 (−1.14 to 0.75) *p =* 0.672	–**4.32 (**–**5.31 to** –**3.05)** ***p** **<*** **0.001**
TNF-α (pg/mL)	Control	8.30 (4.80)	Reference	Reference
	Nutrition	8.10 (5.30)	–**2.10 (**–**3.79 to** –**0.41)** ***p** **=*** **0.017**	–**1.88 (**–**2.94 to** –**0.81)** ***p** **<*** **0.001**
	Nutrition + Exercise	6.50 (1.40)	–**2.30 (**–**3.58 to** –**1.02)** ***p** **<*** **0.001**	–**2.10 (**–**3.26 to** –**1.01)** ***p** **<*** **0.001**

Biomarker data were available for 51 participants (Control, *n* = 20; Nutrition, *n* = 18; Nutrition + Exercise, *n* = 13). ^#^No significant difference (*p* > 0.05) between medians at baseline; ^+^Adjusted for age, sex, ethnicity, education, body mass index, hypertension, hyperlipidemia, diabetes, polypharmacy, sarcopenia, protein intake, intervention compliance, and corresponding baseline values. IL-6, interleukin 6; TNF-α, tumor necrosis factor alpha. Bold indicates significance (*p* < 0.05).

## 4. Discussion

Multidomain interventions incorporating exercise and nutrition have been shown to reverse frailty at the population level and are an important public health priority in many countries (4, 5, 36). Our study demonstrated that 3 months of leucine-enriched protein supplementation and exercise intervention in pre-frail older adults who consumed ≤ 1 g/kg of protein were associated with significant improvement in physical function measures such as gait speed, SPPB, and 5× STS, depression, perceived health, and body composition measures such as FFMI, ASMI, and body cell mass. These were further supported by improvements in inflammatory biomarkers. The Nu group had significant improvements only in body cell mass and inflammatory biomarkers but non-significant improvements in physical function and ASMI. The improvements were not sustained after the discontinuation of the interventions, which suggests a significant contribution of the interventions to the outcomes, especially in the Nu+Ex group. The intake of protein in older adults is often suboptimal, as shown by the 2005–2014 National Health and Nutrition Examination Survey, where 46% of the oldest adults did not meet the requirement of 0.8 g/kg per day (37). Studies have shown that in older adults above 70 years old, lower than 0.8 g/kg per day of protein intake was associated with greater functional limitation and lower HGS (37). Between 2013 and 2014, both the PROT-AGE Study Group and the European Society for Clinical Nutrition and Metabolism (ESPEN) Group recommended increased protein intake of 1.0–1.2 g/kg of body weight per day or higher in older adults, and countries like Singapore have recommended an increase in the RDA of protein for older adults to 1.2 g/kg/day (10, 38, 39).

The amount, quality, type, and timing of protein supplements are known to affect absorption, digestion, and availability. Proteins can be categorized as “fast,” such as whey or soy protein, or slow, such as casein, depending on the speed of release of amino acids. There has been increasing interest in recent years in the use of “fast” proteins to counteract anabolic resistance and improve muscle protein synthesis and muscle function. “Fast” protein in combination with exercise and/or omega-3 fatty acids has been shown to stimulate muscle protein synthesis (40). Studies have shown that peak protein synthesis happens 2 h post-meal, and until recently, the recommendations have been to distribute protein intake across the meals, such as 0.4/kg/meal or 30 g/meal, to counteract anabolic resistance and limit muscle mass loss (41–43). Leucine is recognized as an anabolic stimulus and together with exercise acts through the mechanistic target of the rapamycin complex 1 (mTORC1) pathway in enhancing muscle protein synthesisand immunomodulatory function and reducing muscle protein breakdown (44, 45). Participants in our study received leucine-enriched whey and soy protein, and while we have no information on the timings of leucine-enriched protein supplementation, participants in the Nu+Ex group showed improvement in physical function, inflammation, and body composition measures. Unlike other studies that targeted sarcopenic older adults and supplemented whey protein with vitamin D, leucine-enriched protein supplementation alone did not result in a significant improvement in physical function in our study population (46, 47). The differences between studies could be due to varied study methodology, timing and type of protein intake, type and intensity of exercise, study population, and duration of follow-up (48).

Anabolic resistance with aging is possibly explained by multiple interacting factors such as altered gut microbiome affecting absorption, increased splanchnic sequestration of amino acids affecting peripheral availability, decreased muscle amino acid uptake, lower postprandial perfusion of muscle, and dysfunctional muscle protein synthesis signaling (16). Exercise has been shown to improve the sensitivity of skeletal muscle to dietary protein, and studies have shown that a combination of higher dietary protein and resistance exercise is required for muscle protein synthesis and to counteract anabolic resistance with aging (16). Participants in the Nu+Ex group improved significantly in physical function and muscle mass indices at 3 months.

The benefits of exercise are postulated to be mediated through the activation of the mTORC1 pathway and the release of myokines, osteokines, adipokines, and immune cytokines. The systematic reviews on the benefits of nutrition in addition to exercise have shown mixed results due to varied target groups and nutrition supplements (49). Rondanelli et al. showed that 12 weeks of supplementation with 22 g of whey protein, amino acids including 4 g of leucine, and vitamin D, together with controlled physical activity, increased FFM, physical strength and function, quality of life, and reduced malnutrition in sarcopenic older adults (50). A recent study by Oh et al. showed a leucine-rich protein supplement (20 g protein with 2 g of leucine) together with resistance exercise in healthy older adults improved muscle mass at 12 weeks (11). Recently, a meta-analysis by Negm et al. showed that mixed exercises were most effective in improving muscle mass, whereas physical activity, protein supplementation, and aerobic exercise were most effective in improving physical performance in sarcopenic individuals (51). While the benefit of combined interventions is evident in sarcopenic individuals, studies on the effectiveness of combined interventions in pre-frail or frail participants show mixed results (46, 47, 50, 52). Our study participants participated in mixed exercises biweekly, and the Nu+Ex group showed significant improvement in physical function, FFMI, and ASMI, which was not evident in the control or Nu groups. Various studies have shown that whey protein supplementations of between 22 and 25 g and leucine between 3 and 4 g with vitamin D in combination with exercise intervention increased FFM, relative skeletal muscle mass, and physical function in sarcopenic older adults (50). Pre-frail participants in the Nu+Ex group and Nu group improved in body cell mass. Body cell mass is increasingly recognized as a reliable indicator of muscle mass loss, a measure of metabolically active tissue mass, and nutrition, and is significantly associated with whole body phase angle, which is a significant predictor of mortality (50, 53).

Participants in the Nu+Ex group improved significantly in their GDS scores and perceived health. Berens et al. also showed similar findings from the Vigor 2 study, where combined nutrition and physical activity, but not the nutrition intervention or placebo arm, improved both depression and health-related quality of life (HRQOL) (54). Another study similarly showed physical exercise training and nutritional intervention, but not exercise training alone, over 12 weeks in pre-frail women had beneficial effects on several HRQOL domains (55). Elevated IL-6 has been implicated in depression symptom severity, and 12 weeks of once-weekly exercise sessions have shown a concurrent reduction of depression severity and IL-6 (56). Studies have attributed improvements in depression to exercise due to possible mediation through myokines such as irisin or amino acids like tryptophan which have an antidepressant-like effect (57, 58). It is not known if the improvement in depression in our study participants was mediated through a reduction in IL-6 or through other mechanisms.

Amino acids play a significant role in the proliferation and activation of lymphocytes, natural killer cells, macrophages, and the production of antibodies and cytokines (59). Insufficient dietary protein or amino acid intake has a major impact on the immune system, increases susceptibility to infectious disease, and may exacerbate low-grade inflammation associated with aging (60). Inflammaging, defined as chronic low-grade inflammation with aging, has been associated with many negative outcomes, including insulin resistance, cardiovascular disease, and mortality (60). Most previous studies on the association of protein supplementation with proinflammatory cytokines have focused on healthy older adults, diabetics, or sarcopenic obesity, where the impact on IL-6 and TNF-α has been variable (17, 61, 62). There are as yet no studies on the impact of leucine-enriched protein supplements on IL-6 and TNF-α in pre-frail older adults. Participants in the Nu+Ex and Nu groups showed significant reductions in IL-6 and TNF-α further supporting the role of amino acids in inflammation. Inflammation is common in aging, frailty, and sarcopenia, and it is not known if the improvement in inflammatory cytokines could be mediated through improvements in body composition parameters such as muscle mass or through other mechanisms.

The main strength of this study was robust physical assessments and compliance checking. However, there are several limitations that warrant mention. First, one of the major limitations was the lack of randomization and the significant differences between the control and intervention groups, which were adjusted for in the final analysis. The study was conducted during the COVID-19 pandemic, which interrupted some of the follow-ups and interventions, resulting in variable sample sizes and differences between study populations. Despite the small sample size, significant correlations were obtained, and our results hold clinical value, which requires further validation. Second, chronic disease, 24-h dietary recalls, and functional ability were based on self-reporting, which may be subject to recall bias. Third, there was higher reporting of chronic diseases in the control group, but they had better functional and body composition measures and similar baseline inflammatory biomarkers. This could be due to a recent visit to the doctor for a follow-up on their chronic diseases. The results were adjusted for baseline differences, and benefits were seen only during the intervention period, suggesting a significant contribution of leucine-enriched protein and exercise in the improvement of functional parameters and body composition measures. Fourth, we did not have information on the intake of supplements such as vitamin D and omega-3, which may impact muscle protein synthesis. Fifth, although we advised participants to spread out their protein intake between meals, we have no information on timing, other proteins in daily meals, or the distribution of protein consumption. Our participants mainly consumed a combination of leucine-enriched soy and whey protein. Studies have shown that both are effective in muscle protein synthesis, with no significant differences in strength or muscle mass gain between leucine-enriched soy or whey protein in combination with resistance exercise intervention after 12 weeks (63–65). Sixth, we have no comparison with the exercise-only arm and therefore find it difficult to determine whether the improvements in the Nu+Ex group were due to just the exercise or an interaction between exercise and nutritional supplementation. However, improvement in inflammatory biomarkers was also seen in the Nu group, suggesting a leucine-enriched diet may have a complementary effect. Lastly, due to multiple interacting effects and our study being a quasi-experimental non-randomized study, causal inferences cannot be assumed, and our findings need to be validated in a larger prospective randomized trial. We did not evaluate the palatability and acceptability of the nutrition supplement.

Collectively, data from previous studies in healthy and sarcopenic older adults and our exploratory analysis in pre-frail older adults suggest that leucine-enriched protein supplementation and exercise combination improve physical function, depression, perceived health, body composition such as FFM and body cell mass, and overall inflammation as evident by reduced IL-6 and TNF-α (11, 18, 46–48, 50, 62). To the best of our knowledge, there are limited studies evaluating the benefits of leucine-enriched protein and exercise in terms of physical function, body composition, and inflammation biomarkers in pre-frail older adults with suboptimal baseline protein intake. Our study findings need to be revalidated in future prospective randomized trials targeting at-risk individuals with insufficient protein intake.

Our study demonstrated that additional protein and leucine supplementation together with biweekly exercise over 3 months in pre-frail older adults who consumed ≤ 1 g/kg of protein per day was associated with improvement in gait speed, SPPB, 5× STS, depression, FFMI, and body cell mass on body composition measures and inflammation.

## Data availability statement

The original contributions presented in the study are included in the article/Supplementary material, further inquiries can be directed to the corresponding author.

## Ethics statement

The studies involving human participants were reviewed and approved by National Healthcare Group Domain Specific Review Board. The patients/participants provided their written informed consent to participate in this study.

## Author contributions

RM obtained funding. RM, EP, and KB designed the study. RM, DA, and YC contributed to data analysis and drafting of charts and tables. RM, DA, AL, VN, and YC contributed to the drafting of the manuscript. All authors read, edited, and approved the version submitted.
