# Supplementary material for: Impact of exercise and leucine-enriched protein supplementation on physical function, body composition, and inflammation in pre-frail older adults: a quasi-experimental study
Source: Front Med (Lausanne). 2023 Aug 14;10:1204198. doi: 10.3389/fmed.2023.1204198 (PMC10461448; doi:10.3389/fmed.2023.1204198)
Supplement: Supplementary file 1 [file Table_1.DOCX]

Supplementary Material

Impact of Exercise and Leucine-enriched Protein Supplementation on Physical Function, Body Composition, and Inflammation in Pre-frail Older adults

Reshma Aziz Merchant, Yiong Huak Chan, Denishkrshna.A, Santhosh Seetharaman, Lydia Au, Vidhya Nachammai, Alexa Lai, Vanda Ho, Beatrix Ling Ling Wong, Eunice Pang and Kalpana Bhaskaran

*** Correspondence:** Associate Professor Reshma A Merchant: reshmaa@nuhs.edu.sg

**Supplementary Table 1.** Unadjusted and Adjusted Regression Models of Mean Change in Outcome Variables in Intervention Groups Compared with Control Group

| **Biomarker** | **Group** | **Unadjusted** | **Adjusted^+^** |
| --- | --- | --- | --- |
|  |  | **Coefficient (95% CI)**  **p-value** | **Coefficient (95% CI)**  **p-value** |
| SARC Total | Nutrition | 0.08 (-0.60 – 0.76)  p = 0.820 | 0.42 (-0.40 – 1.24)  p = 0.310 |
|  | Nutrition + Exercise | 0.17 (-0.73 – 1.07)  p = 0.709 | -0.32 (-1.47 – 0.83)  p = 0.582 |
| Perceived Health  (EQ-VAS) | Nutrition | 4.10 (-0.63 – 8.830  p = 0.089 | 0.03 (-6.14 – 6.21)  p = 0.991 |
|  | Nutrition + Exercise | **6.58 (0.24 – 12.93)**  **p = 0.042** | **8.35 (1.41 – 12.50)**  **p = 0.023** |
| Physical Activity (RAPA) | Nutrition | 0.06 (-0.39 – 0.50)  p = 0.055 | -0.20 (-0.75 – 0.36)  p = 0.484 |
|  | Nutrition + Exercise | **0.88 (0.34 – 1.43)**  **p = 0.002** | **0.82 (0.11 – 1.53)**  **p = 0.024** |
| Nutritional Status  (MNA-SF Total) | Nutrition | 0.08 (-0.29 – 0.45)  p = 0.661 | 0.02 (-0.46 – 0.48)  p = 0.933 |
|  | Nutrition + Exercise | 0.13 (-0.36 – 0.61)  p = 0.604 | 0.08 (-0.55 – 0.70)  p = 0.810 |
| Energy Intake | Nutrition | 107.17 (-47.41 – 261.74)  p = 0.172 | -33.35 (-197.46 – 130.77)  p = 0.688 |
|  | Nutrition + Exercise | **332.20 (140.88 – 523.53)**  **p <0.001** | 53.21 (-172.85 – 279.26)  p = 0.641 |
| Protein Intake/Body Weight | Nutrition | **0.27 (0.16 – 0.38)**  **p <0.001** | **0.23 (0.11 – 0.35)**  **p <0.001** |
|  | Nutrition + Exercise | **0.31 (0.17 – 0.44)**  **p <0.001** | **0.25 (0.08 – 0.41)**  **p = 0.003** |

^+^ Adjusted for Age, Gender, Ethnicity, Education, Body Mass Index, Hypertension, Hyperlipidemia, Diabetes, Polypharmacy, Sarcopenia, Protein Intake, Intervention Compliance and corresponding baseline values.

EQ-VAS, EuroQoL Visual Analogue Scale; RAPA, Rapid Assessment of Physical Activity; MNA-SF, Mini Nutritional Assessment-Short Form.
